# 5-Azacytidine-Induced Cardiomyocyte Differentiation of Very Small Embryonic-Like Stem Cells

**DOI:** 10.1155/2020/5162350

**Published:** 2020-09-08

**Authors:** XiaoLin Sun, HongXiao Li, Ye Zhu, Pei Xu, QiSheng Zuo, BiChun Li, Xiang Gu

**Affiliations:** ^1^Institute of Translational Medicine, Medical College, Yangzhou University, Yangzhou 225001, China; ^2^Department of Cardiology, Northern Jiangsu People's Hospital, Yangzhou, Jiangsu 225001, China; ^3^Taizhou People's Hospital, Taizhou, Jiangsu 225300, China; ^4^College of Animal Science and Technology, Yangzhou University, Yangzhou, Jiangsu 225001, China

## Abstract

The use of stem cells in generating cell-based pacemaker therapies for bradyarrhythmia is currently being considered. Due to the propensity of stem cells to form tumors, as well as ethical issues surrounding their use, the seed cells used in cardiac biological pacemakers have limitations. Very small embryonic-like stem cells (VSELs) are a unique and rare adult stem cell population, which have the same structural, genetic, biochemical, and functional characteristics as embryonic stem cells without the ethical controversy. In this study, we investigated the ability of rat bone marrow- (BM-) derived VSELs to differentiate *in vitro* into cardiomyocytes by 5-Azacytidine (5-AzaC) treatment. The morphology of VSELs treated with 10 *μ*M 5-AzaC increased in volume and gradually changed to cardiomyocyte-like morphology without massive cell death. Additionally, mRNA expression of the cardiomyocyte markers cardiac troponin-T (cTnT) and *α*-sarcomeric actin (*α*-actin) was significantly upregulated after 5-AzaC treatment. Conversely, stem cell markers such as Nanog, Oct-4, and Sox2 were continuously downregulated posttreatment. On day 14 post-5-AzaC treatment, the positive expression rates of cTnT and *α*-actin were 18.41 ± 1.51% and 19.43 ± 0.51%, respectively. Taken together, our results showed that rat BM-VSELs have the ability to differentiate into cardiomyocytes *in vitro*. These findings suggest that VSELs would be useful as seed cells in exploring the mechanism of biological pacemaker activity.

## 1. Introduction

Bradyarrhythmias are a group of life-threatening diseases, including sinus syndrome and atrioventricular block, and are characterized by cardiac pacing or conduction dysfunction. Since the late 1950s, bradyarrhythmia therapy has focused on implantation of electronic pacemakers [1]. Although electronic pacemakers are effective, they have several drawbacks and limitations, including infection, metal allergies, electrode dislocation, electronic interference, and lack of neurohormone responsiveness. Biological pacemakers provide an alternative to bradyarrhythmia electronic pacemakers and are generated by somatic gene transfection, cell fusion, or cell transplantation. However, biological pacemakers have been slow to be phased in as a bradyarrhythmia treatment due to the uncertainty of gene transfection targeting and pacing efficiency. Although cellular biological pacemakers have many advantages, there have been difficulties selecting seed cells and induction methods.

Kucia et al. [2] first identified a class of cells in adult mice that maintained pluripotent stem cell activity with cross-dermal differentiation. These cells, known as very small embryonic-like stem cells (VSELs), have been successfully isolated from various tissues from multiple species and contain different surface markers [3–6]. *In vitro* transplantation experiments have shown that VSELs can differentiate into three different embryo layers without forming teratomas following transplantation into the immunodeficient mice [7, 8]. Therefore, VSELs could potentially function as new seed cells for *in vitro* differentiation into a cardiomyogenic, biological pacemaker cell lineage.

The development of a successful cardiomyogenic cell lineage depends on adequate cellular differentiation and proliferation. Cellular differentiation and proliferation are heavily influenced by DNA methylation, and high DNA methylation levels limit these processes. Chemical inhibition of DNA methyltransferases can be effective in overcoming cell growth limitations. 5-Azacytidine (5-AzaC) is a common DNA methyltransferase inhibitor. By covalently binding to DNA methyltransferase, 5-AzaC reduces DNA methyltransferase activity, thereby reducing the methylation level and inducing cell proliferation and differentiation. Several studies have shown that 5-AzaC could induce differentiation of different types of stem cells into cardiomyocytes [4, 8, 9]. Furthermore, these differentiated cells exhibit spontaneous pulsation and measurable action potentials, consistent with cardiomyocytes [4, 8, 9].

In this study, we isolated VSELs from rat bone marrow (BM) and examined whether 5-AzaC treatment could successfully differentiate BM-VSELs into cardiomyocytes. The BM-VSELs retained stem cell characteristics, and treatment with 5-AzaC induced BM-VSEL differentiation into cardiomyocytes. These data provide novel insights into using VSELs and 5-AzaC treatments for building an effective, extracorporeal biological pacemaker.

## 2. Materials and Methods

### 2.1. Isolation and Culturing of BM-VSELs

To obtain a high-purity rat BM-derived population of VSELs, this study adopted the sorting scheme for VSELs proposed by Labedz-Maslowska et al. [4]. All procedures involving the care and use of animals conformed to the Animal Research: Reporting of *In Vivo* Experiments (ARRIVE) guidelines and were approved by the laboratory animal management and experimental animal ethics committee of Yangzhou University.

Bone marrow from 8-12–week-old, male or female, Wistar rats was collected for isolation of VSELs. Rat tibias and femurs were removed and any remaining muscle or connective tissue discarded. The BM was rinsed with phosphate-buffered saline (PBS) (Solarbio, Beijing, China). Total nucleated cells were obtained by lysing red blood cells with 1x BD Pharm Lyse Buffer (BD Pharmingen, San Jose, CA, USA) for 10 min. The nucleated cells were incubated with anti-CD45, anti-CD106, and anti-Lin monoclonal antibodies (1 : 200) at room temperature for 30 min and then sorted by flow cytometry to obtain CD45^−^Lin^−^CD106^+^ VSELs. The following anti-rat antibodies (BD Pharmingen) were used for staining: (i) anti-CD45 (Alexa Fluor 647, clone: OX-1), (ii) FITC-conjugated Lin markers (anti-TCR*αβ* (clone: R73), anti-CD3 (clone: 1F4), anti-CD11b/c (clone: OX-42), and anti-CD45RA (clone: OX-33)), and (iii) anti-CD106 (PE, clone: MR-106).

VSELs were cultured in Dulbecco's Modified Eagle's Medium (DMEM) (Thermo Fisher Scientific, Waltham, MA, USA) containing 10% fetal bovine serum (FBS) (Biowest, Nuaille, France) at 37°C in a 5% CO_2_ incubator. During VSEL expansion and proliferation, culture medium was replaced every three days and cells were passaged after they reached 70% confluency.

### 2.2. Immunofluorescence Staining to Identify VSEL Characteristics

VSELs in healthy growth conditions were plated in 24-well plates with climbing slides. Adherent growth was detected after 24 hours by immunofluorescence staining. VSELs were fixed with 4% paraformaldehyde at 4°C for 30 min. Fixed cells were permeabilized with 0.1% Triton X-100 (Solarbio) and blocked with blocking solution (2% FBS/0.1% Tween 20 (Solarbio)). Fixed cells were incubated overnight at 4°C with primary antibodies (1 : 200 dilution) against rat Nanog, Oct-4, and Sox2 (Proteintech, Chicago, IL, USA). Cells stained with diluent only served as the negative control. After overnight incubation, cells were washed with PBS three times for 5 min each, incubated with CY3-conjugated secondary antibody (1 : 500; Beyotime, Shanghai, China) for 1 hour at room temperature, and washed five times with PBS. Nuclei were stained with 4′,6-diamidino-2-phenylindole (Solarbio). Slides were mounted and examined using a fluorescent microscope (TE2000 Nikon, Tokyo, Japan).

### 2.3. Identification of VSELs by Alkaline Phosphatase Staining

VSELs in healthy growth conditions were plated in 24-well plates with climbing slides, further stained with alkaline phosphatase (AKP) (Solarbio) staining solution, and observed with an inverted microscope (DM1750M Leica, Weztlar, Germany).

### 2.4. Treatment of VSELs with 5-AzaC

VSELs were divided into two separate groups: (1) untreated controls and (2) VSELs treated with various concentrations (1 *μ*M, 10 *μ*M, or 30 *μ*M) of 5-AzaC (Sigma-Aldrich Co., St. Louis, MO, USA). After overnight incubation, the medium was aspirated and cells were washed with PBS three times for 3-5 min. New culture medium was added and subsequently replaced every three days. After 5-AzaC treatment, cell morphology was observed daily using a phase contrast microscope (DMIL-PH1 Leica).

### 2.5. Cell Viability Assay

VSELs were seeded into 96-well plates at 5,000 cells per well. Cells were stained with 100 *μ*L sterile MTT dye (0.5 mg/mL, Solarbio) for 4 hours at 37°C on days 0, 1, 7, 14, and 21 postseeding. Formazan crystals were solubilized with 100 *μ*L DMSO (Solarbio). Absorbance was measured at 490 nm using an automatic microplate reader (Multiskan™ FC, Thermo Fisher Scientific).

### 2.6. Flow Cytometric Analysis of Cardiomyocyte Development

5-AzaC-treated and untreated VSELs were stained for flow cytometric analysis as previously described for characterization of VSELs. Cells were incubated overnight at 4°C with primary antibodies (1 : 200 dilution) against rat-specific cardiac proteins, including cardiac troponin-T (cTnT) (Proteintech) and *α*-sarcomeric actin (*α*-actin) (Proteintech). After overnight incubation, cells were washed with PBS three times for 5 min each, incubated with CY3-conjugated secondary antibody (1 : 500) for 1 hour at room temperature, and washed five times with PBS. The percentage of red fluorescent protein-positive cells was detected by flow cytometric analysis (BD Biosciences, Franklin Lakes, NJ, USA). Untreated VSELs served as a negative control.

### 2.7. RNA Isolation and Reverse Transcription Quantitative Polymerase Chain Reaction (qPCR) Analysis

Using an RNeasy Mini Kit (Qiagen, Dusseldorf, Germany), total RNA was extracted from VSELs treated with 5-AzaC at 0, 7, 14, or 21 days postseeding, according to the manufacturer's instructions. Normal cardiomyocytes were used as the positive control group. cDNA was amplified by PCR using a Qiagen PCR kit according to the manufacturer's instructions. Nanog, Oct-4, Sox2, cTnT, and *α*-actin expression was detected by fluorescent qPCR. The 20 *μ*L PCR amplification reaction included 2 *μ*L cDNA, 10 *μ*L SYBR Taq, 0.8 *μ*L forward primer, 0.8 *μ*L reverse primer, 0.4 *μ*L RoxII, and 6 *μ*L double-distilled water. Each experimental condition was repeated in triplicate. Relative mRNA quantities were determined using the 2^-*ΔΔ*CT^ method.

### 2.8. Statistical Analysis

Data obtained were presented as means ± standard error. Statistical significance was determined using the SPSS 23 statistical program. Variance tests were performed on the data, and GraphPad Prism 7.0 software was used for graphing. The criteria for significance were defined as ∗*p* < 0.05, ∗∗*p* < 0.01, and ∗∗∗*p* < 0.001.

## 3. Results

### 3.1. BM-VSELs Retain Characteristics of Embryonic-Derived Stem Cells (ESCs)

**Figure 1:**
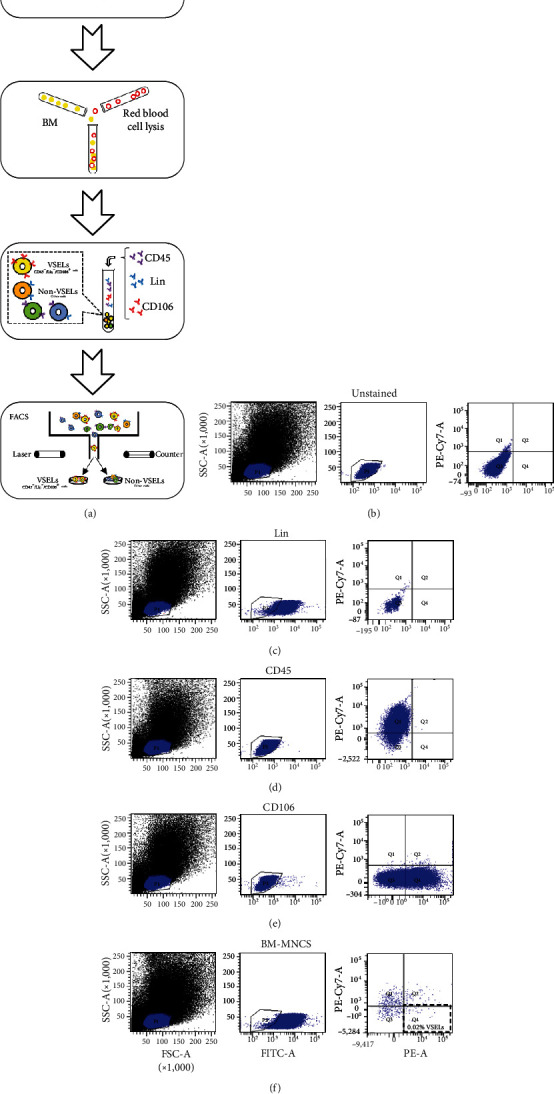
Rat bone marrow- (BM-) derived very small stem cells (VSELs) were successfully isolated by flow cytometry. (a) The tibia and femur of rats were separated, erythrocytes were lysed, and total BM mononuclear cells were extracted. Fluorophore-conjugated antibodies specific to CD45, CD106, and hematopoietic markers (Lin: TCR*αβ*, CD3, CD11b, and CD45RA) were used. Flow cytometric sorting was carried out after incubation. (b) Blank control; (c) Lin control; (d) CD45 control; (e) CD106 control; (f) VSEL sorting.

**Figure 2:**
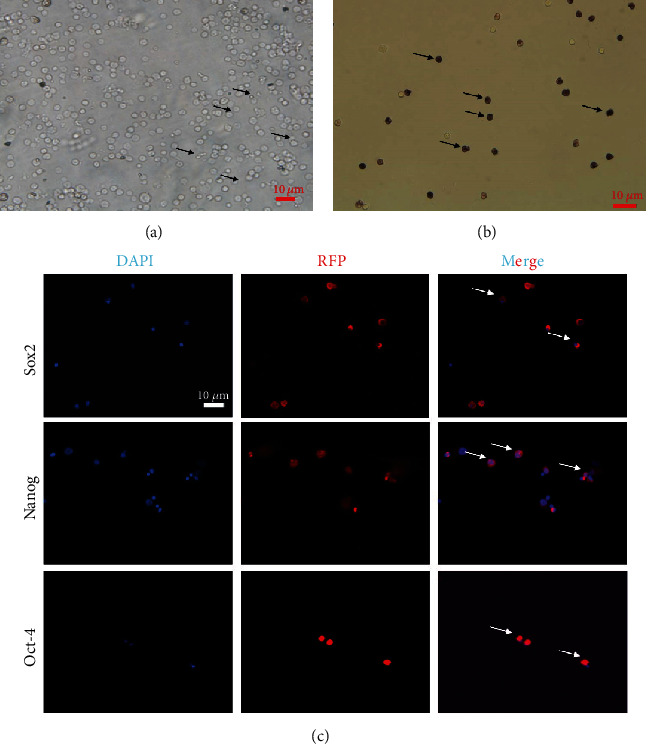
Bone marrow- (BM-) derived very small stem cells (VSELs) retain embryonic stem cell (ESC) characteristics. (a) Rat BM-derived VSELs were obtained by flow cytometric sorting. (b) BM-VSELs were assessed for alkaline phosphatase (AKP) activity by staining for AKP. (c) Expression of Sox2, Oct-4, and Nanog in VSELs was assessed by immunostaining with Sox2-, Oct-4-, and Nanog-specific primary antibodies and a Cy3 secondary antibody.

In this study, VSELs in rat BM were isolated and sorted by flow cytometry. CD45^−^Lin^−^CD106^+^ cells were obtained from BM of adult Wistar rats and defined as VSELs (Figure 1). The majority of VSELs isolated from adult BM are <6 *μ*m, which is larger than peripheral blood platelets, but smaller than erythrocytes (Figure 2(a)). VSELs accounted for 0.020 ± 0.005% of the total number of mononuclear cells (Figure 1), which is consistent with the results from other laboratories [10].

To determine whether BM-VSELs retained the totipotency of ESCs, the expression of totipotency genes Sox2, Oct-4, and Nanog was detected by immunofluorescence. The results showed that VSELs were positive for Sox2, Oct-4, and Nanog (Figure 2(c)), while cardiomyocytes were negative for these proteins. Additionally, AKP activity test is usually used for undifferentiated embryonic stem cells and primordial germ cells, and in our study, AKP staining test showed positive in VSELs (Figure 2(b)). These results indicated that VSELs were successfully isolated from rat BM and retained biological characteristics similar to ESCs.

### 3.2. 5-AzaC Induces Cardiomyocyte-Like Morphology Changes in BM-VSELs

**Figure 3:**
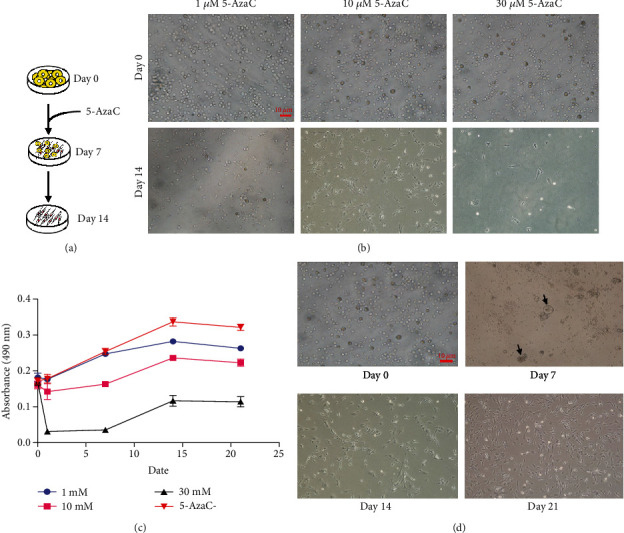
5-AzaC induces cardiomyocyte-like morphology in BM-VSELs. (a) 5-AzaC-induced VSELs showed morphological changes at day 7, and the population of cardiomyocytes peaked at day 14. (b) Growth of VSELs treated with different concentrations of 5-AzaC (1 *μ*M, 10 *μ*M, and 30 *μ*M). (c) MTT assay indicated VSEL growth induced by different concentrations of 5-AzaC over time. (d) Morphological changes in BM-VSELs after treatment with 10 *μ*M 5-AzaC. The arrow indicates that VSELs developed embryoid body-like structures after 5-AzaC treatment.

To investigate the feasibility of 5-AzaC to induce VSEL differentiation into cardiomyocytes, VSELs were treated with 5-AzaC at multiple concentrations and assessed for cardiomyocyte morphology. Without 5-AzaC treatment, there were no obvious morphological changes in the untreated control group (Figure 3(b)). VSELs treated with 1 *μ*M 5-AzaC became slightly more ovular, but did not form the typical cardiomyocyte morphology. Additionally, VSELs treated with 30 *μ*M 5-AzaC showed abnormal cell morphology and enhanced cell death. After exposure to 10 *μ*M 5-AzaC for 24 hours, VSELs were cultured in normal medium. Embryoid body- (EB-) like structures developed in VSELs on day 4 posttreatment. After 5-7 days posttreatment in culture, the cytoplasm gradually stretched to form rod-like structures (Figure 3(d)). Myotube-like morphology was observed in the cultured cells after 14 days posttreatment. At day 21, there was little change in morphology from day 14, and the existing cells began to undergo apoptosis (Figures 3(a) and 3(d)). Cell viability analysis by MTT assay at days 0, 1, 7, 14, and 21 post-5-AzaC treatment revealed that 10 *μ*M yielded the highest number of viable cells. Therefore, 10 *μ*M is the appropriate concentration of 5-AzaC to be used in BM-VSEL treatment (Figure 3(c)).

### 3.3. 5-AzaC Induces BM-VSEL Differentiation into Cardiomyocytes

**Figure 4:**
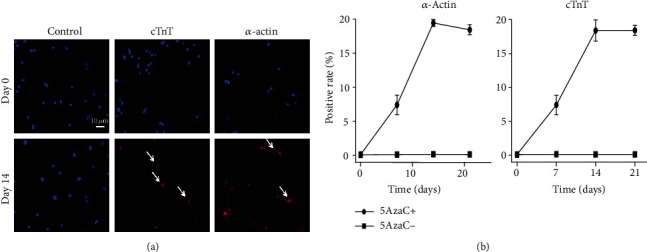
5-AzaC treatment increased levels of cardiac troponin-T (cTnT) and *α*-sarcomeric actin (*α*-actin) in BM-VSELs. (a) Immunostaining for cTnT and *α*-actin in cardiomyocytes derived from 5-AzaC-induced differentiation of VSELs. The white arrow indicates positive cells. (b) Flow cytometric analysis demonstrated the percentages of cTnT- and *α*-actin-positive cells on different days.

To evaluate the differentiation of BM-VSELs into cardiomyocytes, the expression of specific proteins known to be important for cardiomyocyte formation and function was investigated. After 14 days in culture, immunofluorescence staining indicated that BM-VSELs were strongly positive for cardiomyocyte markers, including cTnT and *α*-actin (Figure 4(a)). Conversely, no positive cells were detected in the untreated control group. Furthermore, the flow cytometric analysis revealed that the number of cTnT- and *α*-actin-positive cells began to rise at day 7 and peaked at day 14 posttreatment, showing a significant increase from day 0 (*p* < 0.001). BM-VSEL cTnT- and *α*-actin-positive expression rates reached 18.41 ± 1.51% and 19.43 ± 0.51%, respectively. However, there was no significant difference between days 14 and 21 (*p* > 0.05) (Figure 4(b)). These changes suggest that after exposure to 5-AzaC, the differentiation efficiency of BM-VSELs into cardiac cells was approximately 20%.

### 3.4. 5-AzaC Treatment Reduces Expression of Pluripotent Stem Cell Genes in BM-VSELs

**Figure 5:**
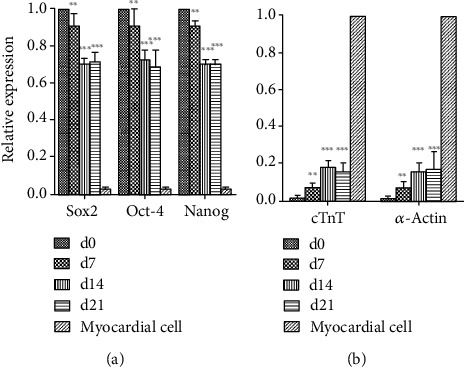
Gene expression levels in BM-VSELs differentiated into cardiomyocytes after 5-AzaC treatment. Expression of (a) Sox2, Oct-4, and Nanog and (b) cTnT and *α*-actin was detected in 5-AzaC-treated cells via qRT-PCR. The gene expression levels of cardiomyocytes and pluripotent stem cell genes were normalized with GAPDH and compared to the relative control group and cardiomyocytes (*n* = 3, level of significance *p* < 0.05; ∗*p* < 0.05; ∗∗*p* < 0.01; ∗∗∗*p* < 0.001).

To evaluate changes in gene expression in VSELs differentiated into cardiomyocytes, we assessed expression of cardiomyocyte-specific and stem cell-specific genes by qPCR. After treatment with 10 *μ*M 5-AzaC, VSELs continued to proliferate and differentiate. The expression levels of cardiomyocyte-specific transcripts cTnT and *α*-actin gradually increased at day 7, with significant differences between day 0 and days 7, 14, and 21 (*p* < 0.05) (Figure 5(b)). The expression levels of stem cell-associated genes Sox2, Oct-4, and Nanog gradually decreased in 5-AzaC-treated VSELs (*p* < 0.05) (Figure 5(a)). The decrease in pluripotent stem cell gene expression and the increase in myocardium-specific gene expression in VSELs after 5-AzaC treatment indicated that VSELs successfully differentiated into cardiomyocytes.

## 4. Discussion

Compared with implantable electronic pacemakers, biological pacemakers are more suitable for treating chronic arrhythmia and pathological sinus syndrome [1]. Moreover, large-animal studies have shown that biological pacing is an effective way to reconstruct the pacing function of sinoatrial node cells [11]. This study was conducted to investigate the ability of BM-VSELs to differentiate into cardiomyocytes after treatment with 5-AzaC *in vitro* to provide sufficient numbers of well-conditioned seed cells for cardiomyocyte differentiation.

ESCs [12], induced pluripotent stem cells (iPSs) [13], and adult stem cells [14–16] are the most common seed cells used for biological pacing. Due to ethical concerns, rejection, tumor-forming tendency, and safety, there are still limitations to their clinical application [17]. Therefore, new biological pacemaker candidate cell types are needed.

VSELs represent a unique and rare population of adult stem cells sharing several structural, genetic, biochemical, and functional properties with ESCs [2]. They have also been identified in several adult murine and human tissues, including ovaries and testicles [4]. VSELs offer several major advantages over currently available seed cells. Given their pluripotent nature and ability to differentiate into cardiomyocytes or endothelial cells, VSELs are particularly well suited for cellular-replacement therapy. The expression of various angiogenic and protective factors in VSELs also renders them suitable for myocardial repair via paracrine actions. Furthermore, unlike the currently available pluripotent cells (ESCs and iPSs), VSELs do not form tumors during extended follow-up studies. Finally, since VSELs can be isolated from adult tissues, the use of autologous VSELs not only avoids rejection and other potential immune risks but also avoids ethical issues [10]. Although VSELs have been isolated from mouse and human tissue for more than a decade, VSELs were not isolated from rat bone marrow until 2016 [4]. Compared with mouse and humans, isolating VSELs from rats is easier and less ethically controversial. In our work, VSELs with high expression of Sox2, Oct-4, and Nanog were successfully isolated from rat BM according to the experimental method of Labedz-Maslowska et al. [4]. VSELs accounted for 0.01-0.02% of the total number of nucleated cells, which was consistent with previous reports [1, 4, 18].

The study by Kassmer et al. [19] confirmed that mouse-derived VSELs can differentiate into lung epithelial cells (endoderm) *in vivo* and Oct-4-positive VSELs have the potential for transdermal differentiation. Mouse BM-VSELs can be differentiated into cardiomyocytes (mesoderm), neurons, astrocytes, oligodendrocytes (ectoderm), and islet cells (endoderm) [20]. Our study may be the first attempt to induce differentiation of rat BM-VSELs into cardiomyocytes.

At present, there are many ways to induce the differentiation of stem cells into cardiomyocytes *in vitro*, including chemical inducers, coculture, conditioned medium of cardiomyocytes [21], and bionic electrical stimulation [22]. However, the chemical inducer and DNA methyltransferase inhibitor 5-AzaC is widely used due to low cost, easy operation, and dosage control. 5-AzaC has been successfully used in the cardiac differentiation of mesenchymal stem cells (MSCs) and human ESCs [23, 24]. The mechanism of 5-AzaC may be related to CpG base-pair demethylation and regulation of early myocardial transcription factors [25–28]. In this study, cultured BM-VSELs were treated with different concentrations of 5-AzaC for 24 hours. The 10 *μ*M 5-AzaC conditions resulted in differentiation of VSELs into cardiomyocyte with fewer apoptotic cells. The concentration of 5-AzaC we used was consistent with the concentration of cardiomyocyte growth induced by Antonitsis et al. in adult bone marrow MSCs [25].

EBs are used as an *in vitro* model for evaluating early extraembryonic tissue formation and differentiation processes. After 48 hours of treatment with 5 *μ*M 5-AzaC, Diomede et al. [29] found that human gingival MSCs (hGMSCs) were organized as round 3D structures (EBs-hGMSCs). In our study, EBs appeared at day 4, later than the hGMSCs induced by Diomede et al. using 5-AzaC, which may be due to the fact that VSELs are a group of inactive heterogeneous cells [30]. After treatment with 5-AzaC, VSELs gradually form muscular tubular structures. Rod-like morphological changes may be related to the expression of proteins that maintain the cytoskeleton.

The increased expression of myocardial-specific proteins and the reduced expression level of pluripotent stem cell genes indicate the transformation of VSELs to cardiomyocytes. It is speculated that genes controlling the differentiation of VSELs into cardiomyocytes are activated and that VSELs successfully differentiate into cardiomyocytes. This may be because 5-AzaC can induce ultrastructural changes in cells, thereby activating key transcription factors and differentiating cells into cardiomyocytes [25]. We found that the number of cTnT- and *α*-actin-positive cells was approximately 20% at day 14, which is similar to 5-AzaC-induced MSC differentiation efficiency [25].

## 5. Conclusion

This study describes the successful isolation of VSELs containing multidirectional differentiation potential from rat BM tissue. Furthermore, the VSELs differentiated into cardiomyocytes through 5-AzaC treatment. These results have important implications for regenerative medicine related to cell therapy of chronic arrhythmias.

## Data Availability

The data used to support the findings of this study are available from the corresponding author upon request.
